# Synchronous angiomyolipoma with epithelial cysts and clear cell renal cell carcinoma: a case report

**DOI:** 10.3389/fonc.2025.1618337

**Published:** 2025-12-04

**Authors:** Jian Wang, Chengde Ren, Ziyang Qiang, Jintao Li, Hailin Ren, Guojun Chen, Xue Gong, Jiaxin Zhang

**Affiliations:** 1 Department of Clinical Medicine, Qinghai University, Xining, Qinghai, China; 2 Department of Urology, Affiliated Hospital of Qinghai University, Xining, Qinghai, China; 3 Department of Pathology, Affiliated Hospital of Qinghai University, Xining, Qinghai, China; 4 Department of Nephrology, Meishan People’s Hospital, Meishan, Sichuan, China

**Keywords:** angiomyolipoma with epithelial cysts, clear cell renal cell carcinoma, angiomyolipoma, epithelial cysts, cystic renal cell carcinoma, renal synchronous tumors

## Abstract

**Background:**

Angiomyolipoma with Epithelial Cysts (AMLEC) is a rare renal tumor that has been established as a separate pathological entity in recent years. Existing literature has focused on its histological origin, molecular features, and imaging characteristics. To our knowledge, this is the first reported case of AMLEC coexisting with clear cell renal cell carcinoma (ccRCC).

**Case presentation:**

A 37-year-old male presented with lumbar pain and persistent gross hematuria. Imaging revealed tumor rupture with hemorrhage, initially suggestive of cystic renal cell carcinoma. However, postoperative pathology and immunohistochemistry confirmed a diagnosis of AMLEC combined with ccRCC.

**Conclusion:**

AMLEC is a rare subtype of angiomyolipoma (AML) that typically lacks adipose tissue, with an epithelial component likely derived from dilated renal tubules. Its unique histological features and immunohistochemical staining are key to pathological diagnosis. This report also reviews prior cases of AMLEC and synchronous renal tumors, offering a valuable reference for clinical diagnosis and management.

## 1 Introduction

Angiomyolipoma with epithelial cysts (AMLEC) is a rare renal tumor recognized as a distinct subtype of angiomyolipoma (AML). Some researchers consider it a cystic variant of AML (1) or a fat-poor type of AML (2, 3). AMLEC was first described by Fine et al. (4) and Davis et al. (5), who referred to it as “angiomyolipoma with epithelioid cyst” and “renal cystic angiomyolipoma,” respectively, with the former term gaining broader recognition.

Due to its distinct histologic morphology and immunophenotype, the Vancouver Classification of Renal Tumors and the International Society of Urological Pathology recognized AMLEC in 2012 as an epithelial cystic variant of AML, though it was not formally categorized as a subtype (6). It was not until 2022 that the World Health Organization (WHO) Classification of Tumors of the Urological and Male Genital System formally classified AMLEC as a subtype of AML (7).

AML consists of mature adipose tissue, smooth muscle, and thick-walled blood vessels (1, 8). In contrast to typical AML, AMLEC is characterized by epithelium-lined cysts, Müllerian tube-like subepithelial stroma, and thick-walled blood vessels, typically lacking adipose tissue (9). Regarding the origin of AMLEC epithelial cells, most researchers (9, 10) support the viewpoint of Fine et al. (4) that the epithelial component of AMLEC is composed of non-tumorous, entrapped, and dilated renal tubules, but this view remains controversial. The most recent study further adds to the findings of Fine et al. that the epithelial and stromal components of AMLEC may represent a hormone-driven proliferation of non-tumorigenic renal components in the context of a dysregulated tumor microenvironment (11).

Clear cell renal cell carcinoma (ccRCC) is the main subtype of renal cell carcinoma, accounting for approximately 70% of all renal cell carcinomas (12). Renal cell carcinoma typically presents as a unilateral lesion; however, the presence of multiple tumors, both synchronous and asynchronous, has been observed in 2–4% of sporadic cases (13). Here, we present a case of a rare synchronous tumor along with its immunohistochemical findings.

## 2 Case report

In September 2023, a 34-year-old male was admitted to our hospital with right-sided lumbar pain, followed by gross hematuria. Physical examination revealed tenderness and percussion pain in the right renal region. There was no bilateral lower extremity edema, and no palpable abdominal mass or evidence of lower back trauma. The patient had no history of chronic diseases, such as hypertension, and no family history of renal cysts, renal tumors, Tuberous Sclerosis Complex (TSC), or related conditions. Before admission, he had consulted multiple medical institutions and was diagnosed with a right renal cyst with hemorrhage.

The patient had not undergone any prior interventions. Following admission, laboratory tests revealed a mildly elevated serum creatinine level of 98 μmol/L (reference range: 57–97 μmol/L), while the complete blood count and other key biochemical markers remained within normal limits.

**Figure 1:**
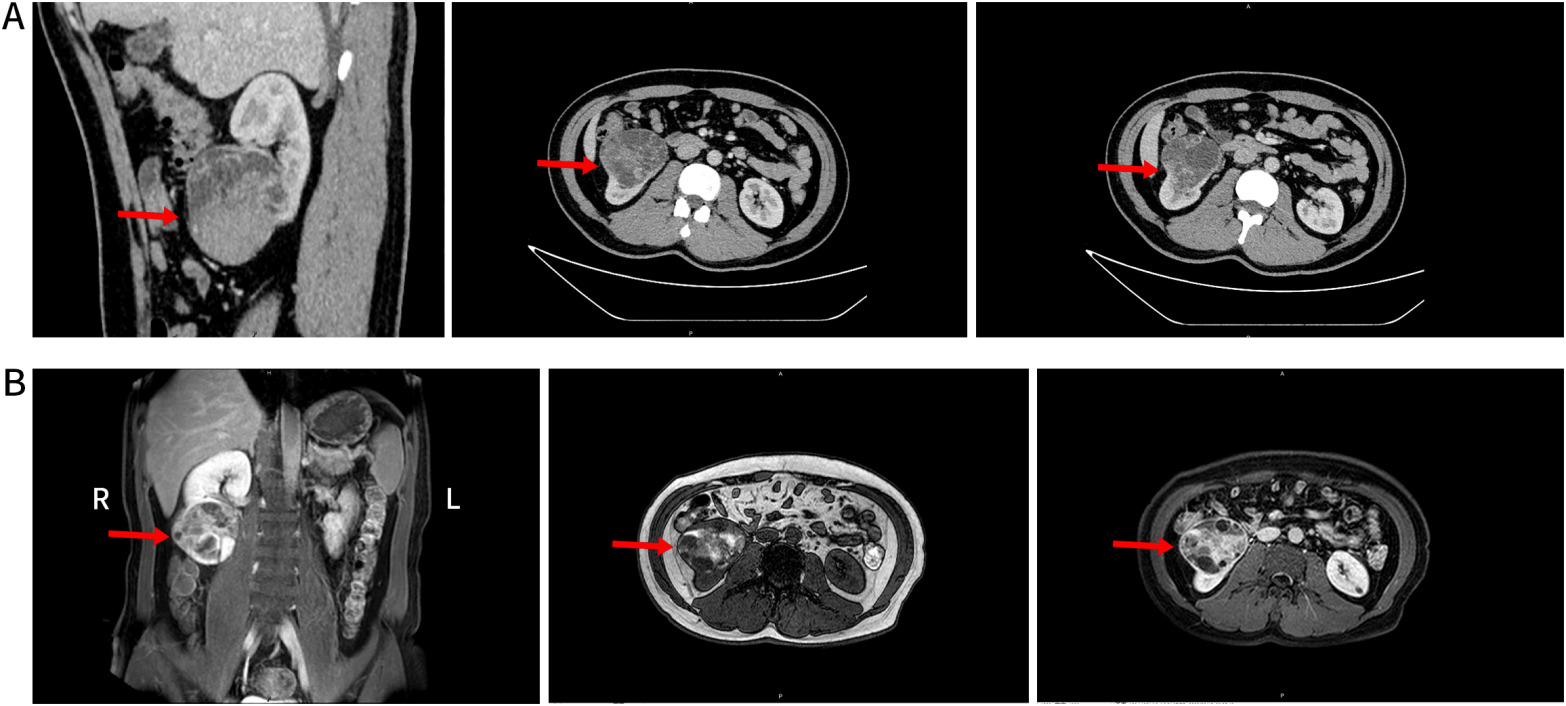
**(A)** Shows the lower pole of the right kidney with an occupying mixed density lesion protruding from the renal silhouette. This lesion had clear borders and a large cross-sectional area of approximately 80×61mm, with uneven density, localized lamellar hyperdense shadows, mild enhancement of the solid portion, and localized lamellar hyperdense shadows with no significant enhancement (red arrows). **(B)** Shows the lower pole of the right kidney and a rounded mass-like mixed lesion with predominantly long T2 and short T1 signals. This lesion protruded outside of the renal silhouette, with a maximum diameter of approximately 75×70×63mm. Multi-cystic changes with segregation were evident inside the lesion, with septal and partial nodular enhancement after enhancement and unclear demarcation between the lesion and the renal pelvis (red arrowheads).

An enhanced computed tomography (CT) scan of both kidneys identified a space-occupying lesion at the lower pole of the right kidney, measuring 80 mm × 61 mm, raising suspicion of malignancy and associated hemorrhage (**Figure 1A**). Similarly, magnetic resonance imaging (MRI) of the lower abdomen detected a lesion in the same region, with internal hemorrhage, measuring 75 mm × 73 mm × 63 mm, and suggested possible encroachment of the renal pelvis (**Figure 1B**).

Urine exfoliative cytology indicated atypical urinary epithelial cells, while urinalysis revealed occult blood at a level of 3 +. Erythrocyte phase contrast microscopy showed homogeneous erythrocyte morphology, thereby excluding a glomerular source of hematuria.

Based on a comprehensive evaluation of the patient’s clinical presentation, imaging findings, and urine exfoliative cytology results, the preliminary diagnosis was a cystic renal cell carcinoma with rupture and hemorrhage. Given the tumor rupture, prominent symptoms, potential for worsening renal function under conservative management, and imaging findings highly suggestive of malignant transformation, surgical intervention was deemed necessary. To preserve renal function, a right ureteral stent was placed preoperatively. On September 19, 2023, under general anesthesia, the patient underwent laparoscopic radical resection of the right kidney and tumor.

Postoperatively, the patient underwent genetic counseling to assess the necessity of TSC gene testing. TSC is an autosomal dominant disorder, with a 50% inheritance risk for offspring of an affected individual (14). Since the patients and their first-degree relatives exhibited neither TSC-related clinical features nor a family history suggestive of TSC, the necessity for TSC1/TSC2 genetic testing was deemed low.

The patient underwent abdominal and chest CT at 3 months, 6 months, and 1 year postoperatively. The patient was in good general condition. Radiological evaluation was consistent with surgical resection and no signs of metastasis were found. Laboratory findings were normal.

## 3 Pathological results

Gross examination revealed a cystic cavity located at the lower pole of the kidney, adjacent to the renal capsule, measuring approximately 70 mm × 60 mm × 20 mm. On sectioning, the lesion showed multiloculated cystic and solid areas and contained bloody fluid.

**Figure 2:**
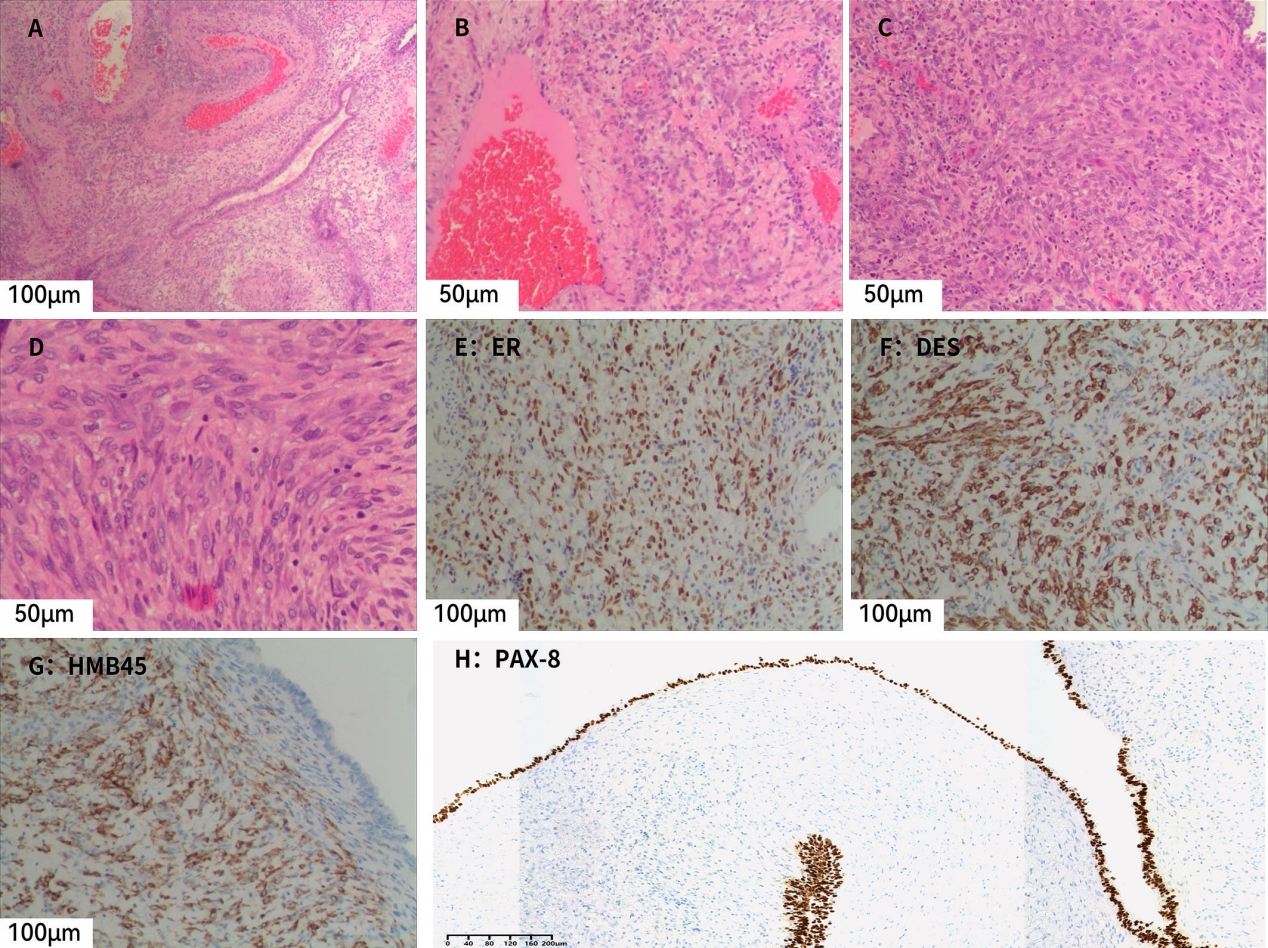
Microscopic and immunohistochemical findings of AMLEC. **(A)** Both epithelial and stromal components are observed, with numerous thick-walled, variably sized dysplastic blood vessels in the surrounding area (H&E, ×20). **(B)** Stromal cells are distributed around blood vessels, with scattered lymphocytes (H&E, ×40). **(C)** Compact stromal cells exhibiting epithelioid or short spindle-shaped morphology (H&E, ×40). **(D)** Stromal cells showing epithelioid features with coarse nuclear chromatin and relatively intact nuclear membranes (H&E, ×40). **(E)** Subepithelial stromal cells show diffuse positivity for ER (×20). **(F)** Subepithelial stromal cells show patchy positivity for desmin (×20). **(G)** Subepithelial stromal cells show diffuse positivity for HMB45 (×20). **(H)** Epithelial cells show diffuse positivity for PAX8 (×10).H&E, hematoxylin and eosin.

At low magnification, cystic structures are evident, with cyst walls of variable thickness, displaying irregular oval and slit-like configurations in some areas. The cysts are lined by a single or multilayered epithelium, beneath which compact stromal cells are observed. At intermediate magnification, the epithelium is composed of a single layer of cuboidal cells or multilayered columnar cells, with focal areas displaying boot-shaped or peg-like projections and focal epithelial denudation. Beneath the epithelium, spindle-shaped, oval, and epithelioid stromal cells are observed, and parts of the stromal component show smooth muscle–like differentiation, with scattered lymphocytes distributed among them. In addition, variably sized, dilated, and congested thick-walled dysmorphic blood vessels are identified (**Figures 2A–D**).

Immunohistochemistry (**Figure 2E–H**) showed that the epithelial component was positive for pancytokeratin (CK) and PAX8, while the stromal component showed positive staining for estrogen receptor (ER), progesterone receptor (PR), CD10, desmin, HMB45, and Melan-A. Smooth muscle actin (SMA) demonstrated focal positivity, and some stromal cells expressed WT-1. Inhibin was negative, and the Ki-67 labeling index was approximately 10%. Based on the immunohistochemical profile, a diagnosis of AMLEC was rendered.

**Figure 3:**
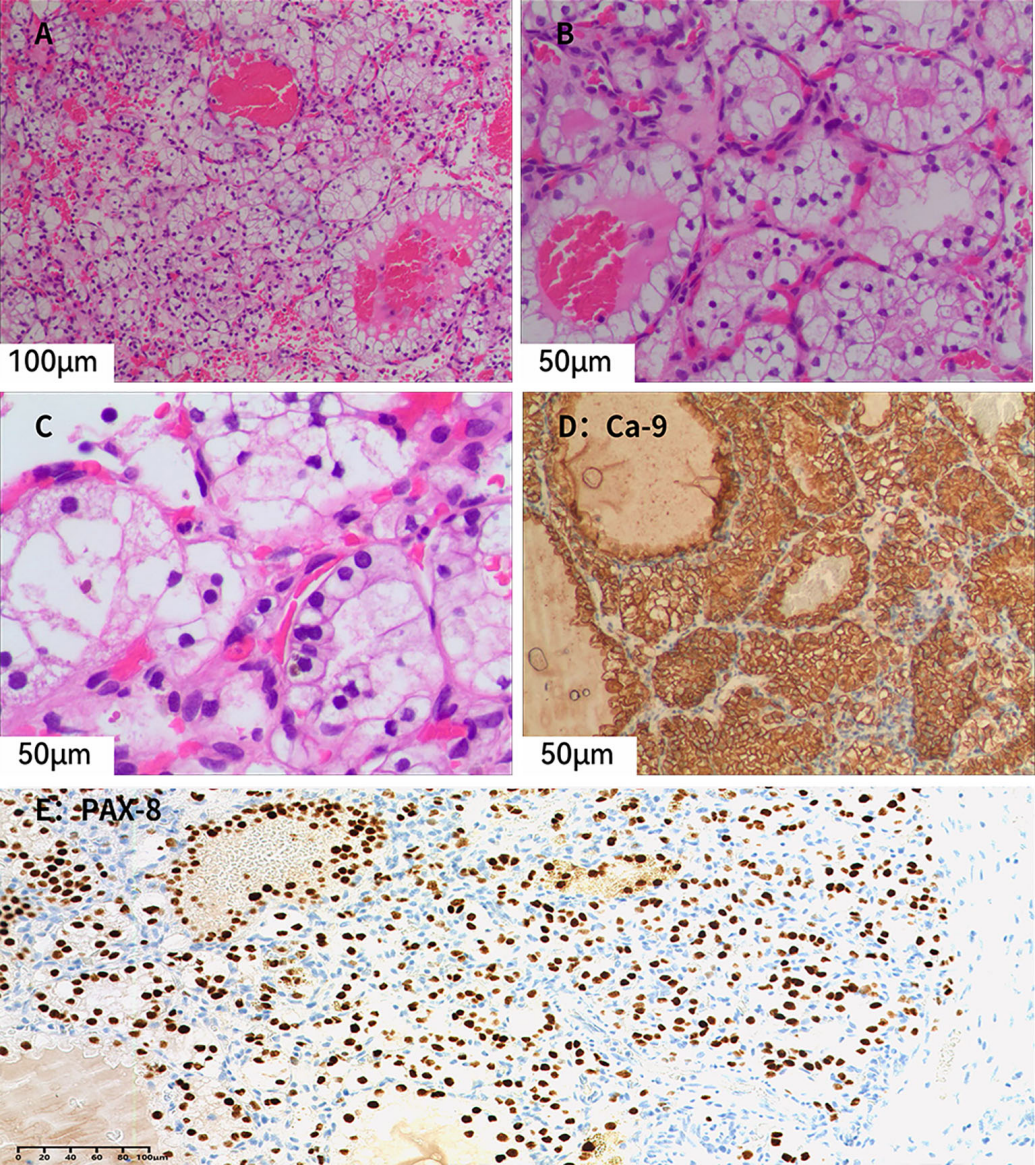
**(A–C)** Microscopic examination reveals tumor cells with distinct cell borders and abundant clear cytoplasm. Small, dilated, and congested capillaries are present within the tumor, and no fibroblastic proliferation is observed (Hematoxylin–eosin staining; **A**, ×20; **B**, ×40; **C**, ×40). **(D)** Tumor cells show diffuse membranous positivity for CA-9 (×40). **(E)** Tumor cell nuclei show diffuse positivity for PAX8, supporting renal epithelial differentiation (×20).

Unexpectedly, a grayish-yellow nodule measuring approximately 3 mm × 2 mm × 0.8 mm, with a golden-yellow cut surface, was identified in the upper pole of the kidney. This nodule had not been detected on preoperative imaging. Microscopically (**Figures 3A–C**), the tumor is well-circumscribed and composed of nests and alveolar structures separated by a delicate vascular network. The tumor cells have abundant clear cytoplasm and centrally located round nuclei. Immunohistochemical analysis (**Figures 3D, E**) showed that the tumor cells were positive for PAX8, CA-9, CD10, and CK7, but negative for CD117 and TFE3. SDHB expression was retained, and the Ki-67 labeling index was approximately 5%. The nodule was diagnosed as ccRCC, staged as pT1aN0M0 and classified as WHO/ISUP grade 1.

Combining all these findings, we finally diagnosed the patient with AMLEC combined with ccRCC. The patient’s treatment timeline is delineated in **Supplementary Figure 1**.

## 4 Discussion

A study of 1,064 cases of postoperative renal angiomyolipoma revealed that only 11 were diagnosed as AMLEC, accounting for approximately 1% of cases (5). Research indicates that AMLEC is more prevalent in males, with a male-to-female ratio of 5:3 (15). The vast majority of patients are clinically asymptomatic, with the condition typically detected incidentally during routine physical examinations or imaging. However, some patients may present with symptoms such as hematuria, proteinuria, an abdominal mass, retroperitoneal bleeding, abdominal pain, groin pain, or renal insufficiency (16, 17). When patients present with gross hematuria and lumbar pain, this may suggest an emergent clinical condition, such as bleeding from a ruptured tumor.

Classic AML is readily identifiable on CT and MRI due to its abundant mature adipose tissue. In contrast, AMLEC typically lacks adipose content and exhibits cystic features, which Acar et al. described as a rare variant of fat-poor angiomyolipoma (2). Since most previously reported cases of AMLEC fall under Bosniak type III or IV and exhibit imaging characteristics similar to malignant cystic renal tumors, such as cystic renal cell carcinoma (cRCC) and cystic nephroma (CN), they are frequently misdiagnosed preoperatively as other renal neoplasms (2, 18–20). Some studies suggest that contrast-enhanced ultrasound (CEUS) may be a promising additional diagnostic tool that not only demonstrates good performance in differentiating benign and malignant renal lesions, but also improves the diagnostic sensitivity of malignant cystic masses (21–24).

AMLEC displays distinct immunohistochemical features that assist in its diagnosis. The cyst-lining epithelium expresses PAX8, PAX2, and CK7, while the subepithelial stromal region shows strong positive staining for ER, PR, CD10, Melan-A, and HMB45. Additionally, the muscular component stains most intensely for smooth muscle actin and desmin (25).

The role of renal puncture biopsy in the early diagnosis and risk stratification of renal cystic tumors remains a topic of ongoing debate (26). Some researchers argue that biopsy may cause complications, such as hemorrhage or tumor dissemination, and therefore should not be employed routinely (27). Conversely, given the challenges of definitively identifying fat-poor angiomyolipomas using CT or MRI alone, some advocate for percutaneous biopsy in such cases (28–30). Moreover, in TSC-associated renal cystic tumors, biopsy may induce hemorrhage in highly vascularized angiomyolipomas or even facilitate malignant cell dissemination, making it generally inadvisable (31). Overall, the clinical indications for renal puncture biopsy require further investigation.

Recent reports have explored the molecular basis of AMLEC. Xu et al. (32) identified a TSC1 splicing mutation in a fat-poor AMLEC, while Song et al. (33) reported a TSC2 nonsense mutation associated with activation of the PI3K–AKT–mTOR pathway. These findings highlight the potential for molecular diagnosis of AMLEC in clinical practice. However, given the sporadic reports, further studies are needed to validate these mutations and assess their role in guiding potential targeted therapies.

An intriguing aspect of AMLEC is its relationship with TSC. AML occurs in approximately 80% of adult patients with TSC and typically manifests as unilateral or bilateral multifocal lesions (34, 35). However, the relationship between AMLEC and TSC remains unclear, with only three reported cases of TSC co-occurring with AMLEC in the literature (4, 10, 36). Thus, there is no evidence that AMLEC is a lesion exclusive to TSC, with the majority of cases appearing to be sporadic (19).

In this case, ccRCC and AMLEC were identified in the upper and lower poles of the right kidney, respectively, occupying distinct anatomical regions. This necessitates differentiation among collision tumors, composite tumors, and synchronous tumors (37). Collision tumors are defined as the simultaneous presence of multiple tumors of different types in the same part of the same kidney, which are histologically distinct in origin and non-confluent. Composite tumors consist of two morphologically and immunohistochemically distinct tumor types within the same mass, often representing divergent differentiation of a single neoplasm. In contrast, synchronous tumors refer to the presence of two different tumors diagnosed simultaneously or within a short time frame, with well-demarcated borders, occurring in separate regions of the same organ or in different anatomical sites. Therefore, based on the above definitions, they were identified as synchronous renal tumors.

Synchronous renal tumors have been widely reported; however, synchronous AMLEC tumors are exceptionally rare, with only five cases documented to date (**Table 1**). To our knowledge, this case represents the sixth reported instance and the first documented occurrence of a synchronous tumor involving both AMLEC and ccRCC. Previous studies have described synchronous tumors comprising AML and ccRCC, which are classified as rare clinical entities (38). Additionally, Sakr et al. (39) noted that synchronous but histologically distinct in origin ipsilateral renal tumors constitute a rare phenomenon. The synchronous tumors in this case have different histological origins, and their prognosis and clinical management are challenging.

**Table 1:** Previously reported cases of synchronous tumors involving AMLEC. .

NO.	Authors	Year reported	Age(years)	Gender	Left/Right	Maximum tumor diameter (cm)	Fat content	Treatment	TSC	Coexisting renal tumor type	Histological relationship
1	Fine et al. (4)	2006	37	Female	Left	1.3	A few	Radical nephrectomy	Yes	Papillary renal cell carcinoma	Different
2	Davis et al. (5)	2006	70	Male	Right	4	None	Partial nephrectomy	No	Papillary adenomas	Different
3	Davis et al. (5)	2006	61	Female	Left	6	None	Radical nephrectomy	No	Papillary renal cell carcinoma	Different
4	Takei et al. (40)	2016	49	Male	Right	4.5	A few	Radical nephrectomy	No	AML	Same
5	Cho et al. (36)	2019	46	Female	Left	3	Unknown	Partial nephrectomy	Yes	ESC-RCC	Different
6	Present case	2025	36	Male	Right	8	None	Radical nephrectomy	Unknown	ccRCC	Different

AML, angiomyolipoma; ccRCC, clear cell renal cell carcinoma; ESC-RCC, eosinophilic solid and cystic renal cell carcinoma; TSC, tuberous sclerosis complex.

Solitary AMLEC exhibits favorable biological behavior, with no reported cases of recurrence or metastasis. Surgical intervention, including nephron-sparing surgery and radical nephrectomy, is the mainstay of treatment for large or symptomatic AMLEC, as demonstrated in our case (27). For patients with asymptomatic or tumors less than 4 cm in diameter, close follow-up observation is a viable option. When tumor diameter exceeds 4 cm, either surgical resection or selective arterial embolization may be considered to mitigate the risk of hemorrhage due to tumor rupture (20, 32). Additionally, for TSC-associated renal AMLEC, mTOR inhibitors may serve as a therapeutic option (41).

In cases where AMLEC coexists with malignant lesions such as ccRCC, treatment should prioritize the malignant component. For synchronous ipsilateral renal tumors, nephron-sparing surgery achieves tumor-specific survival rates comparable to those of radical nephrectomy (42, 43). While partial nephrectomy and focal ablation therapies offer advantages in preserving renal function, radical nephrectomy remains a reasonable option for complex or indistinguishable lesions. The surgical approach should be individualized based on the size, location, and presumed pathology of the synchronous tumors (44). Given the limited long-term data on synchronous renal tumors, further research is warranted to refine treatment strategies and evaluate disease progression in these rare cases.

## 5 Conclusions

AMLEC is a rare subtype of AML rather than a primary cystic lesion. Due to its lack of distinctive clinical and imaging features, preoperative diagnosis is challenging, with definitive diagnosis relying on postoperative histopathological examination and immunohistochemical analysis. Future research should further investigate the role of TSC1/TSC2 gene mutations in AMLEC to elucidate its molecular mechanisms and clinical significance in its pathogenesis. This study underscores the importance of managing rare cystic renal tumors and highlights the need for accurate differentiation between collision tumors, composite tumors, and synchronous tumors during diagnosis and treatment.

This study has certain limitations. The patient did not undergo TSC1/TSC2 genetic testing, which to some extent limited our ability to further analyze the molecular characteristics of AMLEC. Future investigations should prioritize targeted TSC1/TSC2 profiling to elucidate the potential contribution of TSC-pathway alterations to AMLEC pathogenesis.

## Data Availability

The original contributions presented in the study are included in the article/**Supplementary Material**. Further inquiries can be directed to the corresponding author.

## Supplementary material

Supplementary Figure 1 Timeline of the patient’s treatment.
