# Supplementary figures and images for: Synchronous angiomyolipoma with epithelial cysts and clear cell renal cell carcinoma: a case report
Source: Front Oncol. 2025 Dec 4;15:1618337. doi: 10.3389/fonc.2025.1618337 (PMC12711486; doi:10.3389/fonc.2025.1618337)

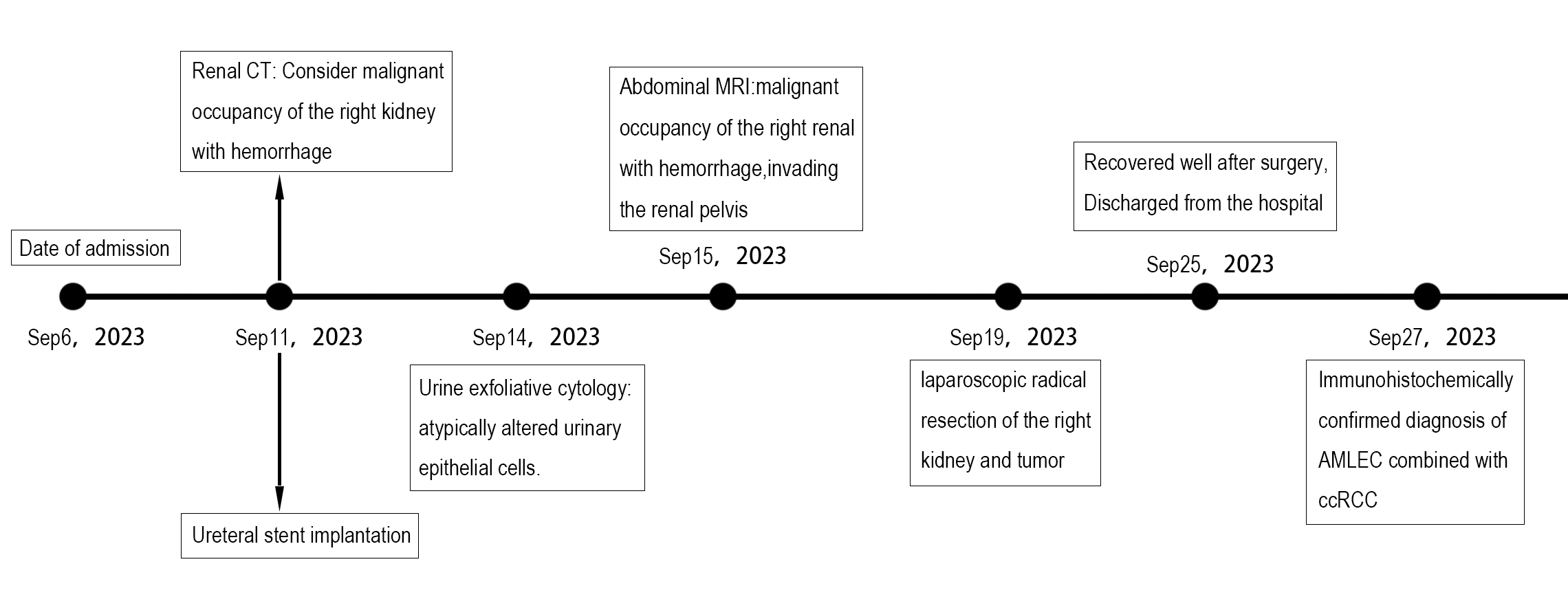

Supplement: Supplementary Figure 1 — Timeline of the patient’s treatment. [file Image1.tif]
